# The Association Between Breast Cancer and Blood-Based Methylation of **S100P** and **HYAL2** in the Chinese Population

**DOI:** 10.3389/fgene.2020.00977

**Published:** 2020-08-28

**Authors:** Qiming Yin, Xiaoqin Yang, Lixi Li, Tian Xu, Wenjie Zhou, Wanjian Gu, Fei Ma, Rongxi Yang

**Affiliations:** ^1^Department of Epidemiology and Biostatistics, School of Public Health, Nanjing Medical University, Nanjing, China; ^2^Department of Breast Surgery, West China Hospital, Sichuan University, Chengdu, China; ^3^Department of Medical Oncology, National Cancer Center/National Clinical Research Center for Cancer/Cancer Hospital, Chinese Academy of Medical Sciences & Peking Union Medical College, Beijing, China; ^4^Department of Clinical Laboratory, Jiangsu Province Hospital of Chinese Medicine, Nanjing, China; ^5^Chengdu Shang Jin Nan Fu Hospital, West China Hospital, Sichuan University, Chengdu, China

**Keywords:** epigenomics, breast cancer, DNA methylation, S100 calcium-binding protein P gene, hyaluronoglucosaminidase 2 gene

## Abstract

Previous work has shown that DNA methylation in peripheral blood may be associated with malignancy; however, these studies have mainly been conducted within Caucasian populations. Here, we investigated the association between blood-based methylation of S100 calcium-binding protein P gene (*S100P*) and hyaluronoglucosaminidase 2 gene (*HYAL2*) and breast cancer (BC) via mass spectrometry in two independent case-control studies of the Chinese population with a total of 351 BC cases and 427 cancer-free female controls. In Study I, in which subjects had an average of 45 years, hypomethylation of *S100P* showed a protective effect for women ≤45 years (six out of nine CpG sites, *p* < 0.05) but not for women >45 years. In contrast, hypomethylation of *HAYL2* was not correlated with BC in women ≤45 years but was a risk factor for women >45 years (three out of four CpG sites, *p* < 0.05). We proposed an age-dependent correlation between BC and methylation of *S100P* and *HYAL2* and performed further validation in Study II with older subjects (average age = 52.5 years), where hypomethylation of both *S100P* and *HYAL2* was a risk factor for BC (*p* < 0.05 for 10 CpG sites) as reported in Caucasians who develop BC around 55 years old. Together with the observation that Chinese cancer-free females having variant basal methylation levels comparing to Caucasians, we assumed that blood-based methylation might be modified by ethnic background, hormone status, and lifestyle. Here, we highlighted that the epigenetic biomarkers warrant validations when its application in variant ethnic groups is considered.

## Introduction

As the most common cancer among women, breast cancer (BC) resulted in 2.1 million new cases and 626,679 deaths in 2018 according to the estimation of WHO (Bray et al., 2018). As the outcome of patients with primary BC is closely correlated with the number of involved axillary nodes and tumor size, the early detection of BC is necessary and appreciated. BC is a complex and heterogeneous disease caused by both genetic and non-genetic factors. Female gender, age, and family history are the major risk factors for BC (Benson et al., 2009). However, even combining those three major factors with other known factors, such as lifestyle, exposure to hormones, and medical and reproductive factors, the predictive model for BC has an accuracy of just 58–59% (Decarli et al., 2006). Genome-wide association studies have identified multiple variants with low-penetrance risk to BC, but a model of 10 single-nucleotide polymorphisms (SNPs) still only reached a predictive accuracy of 59.7% (Wacholder et al., 2010). Thus, there is still a lack of biomarkers for the evaluation of BC risk, especially for the early detection of BC.

Epigenetics is defined as changes in gene expression that are not caused by alterations in the sequence of DNA. DNA methylation is a covalent modification that occurs exclusively on cytosine nucleotides and almost always in the context of CpG. Regulation systems write the methylation pattern *de novo* by removing methyl groups and copying methylation patterns during the DNA replication of cells. As one of the most important components of epigenetics, DNA methylation represents an expression system of genes via controlling how and when to read the information and to initiate the transcription (Domcke et al., 2015). Recent studies revealed that the pattern of DNA methylation varies with aging (Horvath, 2013; Nejman et al., 2014), imprinting genetic disease, such as transient neonatal diabetes mellitus, Silver–Russell syndrome (Elhamamsy, 2017), and cancer (Esteller, 2011; Feinberg et al., 2016). In cancer, the epigenetic events modulate gene expressions either by inhibition of tumor suppressor genes or by activation of oncogenes (Feinberg et al., 2006). Thus, even if DNA methylation may not play a dominant role in all cancer types, there is no doubt that these modification patterns significantly affect cell predisposition and tumor phenotypes. As an early event of carcinogenesis, DNA methylation also shows emerging clinical applications on the diagnosis of cancer. Recent studies between cancer and DNA methylation in blood mainly mostly focused on cell-free DNA or circulation tumor DNA, but so far showed limited contribution for the diagnosis of early stage cancer. Chen et al. (2018) reported that the change of methylation patterns of DNA abstracted from leukocytes occurred earlier than the appearance of physical changes, such as infrequent glucose level alternation and dynamic changes during viral infections. Leukocytes may play a critical role in the initiation, angiogenesis, progression, invasion, and metastasis of cancer (Coffelt et al., 2015; Lopez-Soto et al., 2017). Thus, there is a possibility that the DNA methylation pattern in leukocytes may change along with the cancer progresses. In our previous study, we observed BC-associated S100 calcium-binding protein P gene (*S100P*) and hyaluronoglucosaminidase 2 gene (*HYAL2*) hypomethylation in the blood cells of the Caucasian population (Yang et al., 2015, 2017). Since DNA methylation pattern might be influenced by different ethnic backgrounds and numerous environmental factors (Marks et al., 2004; Zhang et al., 2011; Bind et al., 2014; Elliott et al., 2014; Panni et al., 2016; Sapienza and Issa, 2016; Martin and Fry, 2018), it would be meaningful to investigate the association between blood-based *S100P* and *HYAL2* methylation and BC in another ethnic group, such as in the Chinese population.

## Materials and Methods

### Study Population

This study was approved by the ethics committees of Nanjing Medical University, the Chinese Academy of Medical Sciences, and West China Hospital. All the recruited cases and controls gave written informed consent.

#### Study I

Two hundred eighty-seven sporadic BC cases with a median age of 45 years (34–73 years old) were collected from the Cancer Hospital of Chinese Academy of Medical Sciences from 2015 to 2018. All the patients were confirmed and staged by pathologic results after surgery. Cancer-free females were recruited from the Health Center in Jiangsu Province Hospital of Chinese Medicine. A total of 332 unrelated females were randomly selected during the year of 2018 as controls. All the female controls were self-report healthy, no cancer history, no autoimmune diseases, and with normal blood counts. No further inclusion criteria were applied for the controls. The median age of the healthy controls was 45 years (range from 25 to 78 years).

#### Study II

Sixty-four sporadic BC cases with a median age of 52.5 years (22–77 years old) were collected before surgery and before any BC-related treatment from West China Hospital in 2018. All the patients were confirmed and staged according to pathologic results after surgery. A total of 95 unrelated healthy females were randomly recruited from the same Health Center in Jiangsu Province Hospital of Chinese Medicine during the year of 2018. The recruit criteria are described as above. The median age of the controls for Study II was 46 years (28–78 years).

### Sample Collection and Processing

According to our unpublished data, the temperature of blood at processing influences the levels of DNA methylation. Thus, peripheral whole blood was collected by ethylenediaminetetraacetic acid (EDTA) tubes and kept at 4°C within 8 h before being stored at −80°C until further usage. Genomic DNA was isolated from peripheral whole blood using the Genomic DNA Extraction Kit (Rebece, Nanjing, China). DNA was bisulfite converted by EZ-96 DNA Methylation Gold Kit according to the standard protocol (Zymo Research, Orange County, United States).

### Matrix-Assisted Laser Desorption/Ionization-Time-of-Flight Mass Spectrometry

Agena matrix-assisted laser desorption/ionization (MALDI)-time-of-flight (TOF) mass spectrometry (Agena Bioscience, San Diego, California, United States) described by Yang et al. (2015, 2017) was used to determine the levels of DNA methylation semiquantitatively. In short, the bisulfite-converted DNA was amplified by bisulfite-specific primers (Supplementary Figure 1). Neither the primers nor the amplicons were overlapped with any known SNPs. The PCR products were treated according to the standard protocol of Agena EpiTyper Assay and further cleaned by resin and then dispensed to a 384 SpectroCHIP by a Nanodispenser. The CHIP was read by a MassARRAY system. Data were collected by EpiTYPER v1.2 software. The samples from BC cases and controls were treated and analyzed in parallel in all the processes. Meanwhile, the same amounts of cases and controls were analyzed on each chip for the analyses of MassARRAY.

### Statistical Analyses

All the statistical analyses of MassARRAY data were conducted by SPSS22.0. Between-group differences in age were calculated by *t*-test. The correlations were assessed by Spearman’s rank correlation coefficients. Logistic regression models were used for comparison between groups and adjusted for possible confounding effects by including additional covariables into the models. Non-parametric tests were used to test whether the DNA methylation levels of *S100P* and *HYAL2* differed between different clinical features and to calculate the trend tests of risk or protective effects of methylation. All statistical tests were two-sided, and *p* < 0.05 were considered statistically significant.

## Results

### Breast Cancer-Associated Hypermethylation of S100P in Study I

To investigate the BC-associated differential *S100P* and *HYAL2* methylation in the peripheral blood DNA of the Chinese population, the same amplicons as described by Yang et al. (2015, 2017) including a total of 13 CpG sites were amplified and analyzed by Agena MALDI-TOF mass spectrometry in 287 sporadic BC cases and 332 age-matched female cancer-free controls.

**FIGURE 1:**
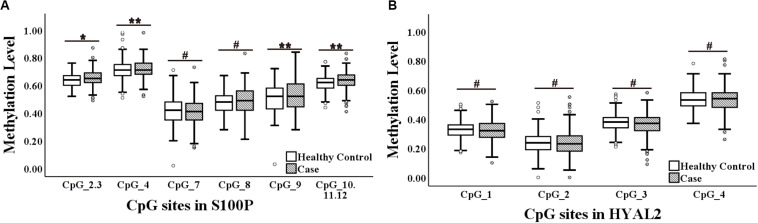
Methylation levels of CpG sites in S100 calcium-binding protein P gene (*S100P*) and hyaluronoglucosaminidase 2 gene (*HYAL2*) in sporadic breast cancer (BC) cases and controls in Study I measured by MassARRAY. **(A)** Box plot shows the distribution of methylation levels of all nine measured CpG sites in *S100P*. **(B)** Box plot shows the distribution of methylation levels of all four measured CpG sites in *HYAL2*. The *p*-values were calculated by logistic regression adjusted for age and batches of measurement; ^#^*p* > 0.05, *p ≤ 0.05, **p ≤ 0.01, ***p ≤ 0.001.

The current study showed an association between increased methylation of *S100P* and BC (S100P_CpG_2.3, S100P_CpG_4, S100P_CpG_9, and S100P_CpG_10.11.12; all *p* ≤ 0.05; Figure 1A), which is contrary to the previous report in Caucasians that BC was associated with decreased methylation of *S100P* (Yang et al., 2017). More specifically, hypomethylation of each CpG site in *S100P* showed variant protective effect to BC, among which S100P_CpG_9 was the most significant loci with an odds ratio (OR) of 0.64 per 10% decrease of methylation (95% CI = 0.48–0.85, *p* = 0.002; Table 1), whereas OR per 10% decrease of methylation for S100P_CpG_2.3 was 0.59 (95%CI = 0.38–0.91, *p* = 0.017), for S100P_CpG_4 was 0.60 (95% CI = 0.42–0.84, *p* = 0.003), and for S100P_CpG_10.11.12 was 0.61 (95% CI = 0.44–0.84, *p* = 0.003; Table 1). Although not significant, S100P_CpG_7 and S100P_CpG_8 also showed ORs < 1.00 per 10% decrease of methylation (Table 1).

**TABLE 1:** Methylation differences of *S100P* and *HYAL2* between BC cases and controls in Study I.

**CpG sites**	**Controls median (IQR)**	**BC cases median (IQR)**	OR (95% CI)* per −10% methylation	*p*-value*
S100P_CpG_2.3	0.64 (0.60–0.67)	0.65 (0.62–0.69)	0.59 (0.38–0.91)	**0.017**
S100P_CpG_4	0.71 (0.67–0.75)	0.71 (0.68–0.76)	0.60 (0.42–0.84)	**0.003**
S100P_CpG_7	0.42 (0.35–0.48)	0.41 (0.35–0.47)	0.95 (0.78–1.16)	0.601
S100P_CpG_8	0.48 (0.43–0.53)	0.49 (0.42–0.56)	0.97 (0.77–1.23)	0.792
S100P_CpG_9	0.52 (0.43–0.58)	0.52 (0.44–0.61)	0.64 (0.48–0.85)	**0.002**
S100P_CpG_10.11.12	0.62 (0.58–0.65)	0.64 (0.60–0.68)	0.61 (0.44–0.84)	**0.003**
HYAL2_CpG_1	0.33 (0.29–0.37)	0.32 (0.28–0.37)	1.00 (0.73–1.37)	0.984
HYAL2_CpG_2	0.24 (0.19–0.28)	0.23 (0.18–0.29)	0.96 (0.73–1.24)	0.730
HYAL2_CpG_3	0.38 (0.34–0.41)	0.37 (0.32–0.41)	1.19 (0.83–1.71)	0.343
HYAL2_CpG_4	0.53 (0.49–0.58)	0.54 (0.48–0.58)	0.90 (0.68–1.20)	0.474

*Logistic regression, adjusted for age and batches of measurement. BC, breast cancer; S100P, S100 calcium-binding protein P gene; HYAL2, hyaluronoglucosaminidase 2 gene; OR, odds ratio; IQR, interquartile range. The bold values indicate p < 0.05.

Interquartile analyses (Flanagan et al., 2009) were also carried out to evaluate the ORs of methylation levels in all the *S100P* CpG sites to the risk of BC. Comparing to the quartile with the highest methylation level (Q4), S100P_CpG_2.3, S100P_CpG_8, S100P_CpG_9, and S100P_CpG_10.11.12 showed significant association with decreased risk of BC in most of the lower quartiles of methylation levels (Q1–Q3) (*p* < 0.05 by logistic regression adjusted for age and batches of the measurement; Supplementary Table 1). And this association was especially pronounced in the lowest quartile (OR for Q1 of S100P_CpG_2.3 = 0.52, *p* = 0.005; OR for Q1 of S100P_CpG_8 = 0.61, *p* = 0.041; OR for Q1 of S100P_CpG_9 = 0.53, *p* = 0.007; OR for Q1 of S100P_CpG_10.11.12 = 0.43, *p* = 3.00E-4; Supplementary Table 1). Moreover, the protective effects of methylation in S100P_CpG_2.3, S100P_CpG_9, and S100P_CpG_10.11.12 were enhanced with lower quartiles (*p*_*trend*_ for S100P_CpG_2.3 = 0.003, *p*_*trend*_ for S100P_CpG_9 = 0.041, and *p*_*trend*_ for S100P_CpG_10.11.12 = 2.00E-4; Supplementary Table 1).

In *HYAL2*, we did not observe any correlation between all four CpG sites and BC in Study I either by direct logistic regression analyses or by interquartile analyses (Figure 1B, Table 1, and Supplementary Table 2).

### Age-Correlated S100P and HYAL2 Methylation in Study I

As age played a crucial role in DNA methylation (Horvath, 2013; Nejman et al., 2014), we calculated the correlation between age and methylation levels in controls and BC cases in Study I. All the nine methylation sites of *S100P* showed significant correlation with age in the controls (Spearmen rhos range from 0.15 to 0.24, *p*-values range from 9.00E-6 to 0.006; Supplementary Table 3), whereas seven methylation sites of *S100P* showed significant correlation with age in BC cases (Spearmen rhos range from 0.13 to 0.38, *p*-values range from 7.3E-11 to 0.025; Supplementary Table 3). In the gene of *HYAL2*, HYAL2_CpG_4 was correlated with age only in the controls (Spearmen rho = 0.18, *p* = 0.037; Supplementary Table 3), whereas HYAL2_CpG_2 and HYAL2_CpG_3 were inversely correlated with age only in the BC cases (*p* < 0.05; Supplementary Table 3).

Since both *S100P* and *HYAL2* showed differential age-related patterns in controls and BC cases, we further stratified the subjects in Study I by the median age of 45 years. In the group of younger than 45 years (including 45 years), hypomethylation of all the nine *S100P* CpG sites showed protective effects to BC (ORs < 1.00 for all; Table 2). Among which the correlation between BC and decreased methylation of S100P_CpG_2.3, S100P_CpG_9, and S100P_CpG_10.11.12 was significant (per −10% of methylation, OR = 0.46 for S100P_CpG_2.3, OR = 0.61 for S100P_CpG_9, and OR = 0.55 for S100P_CpG_10.11.12; all *p* < 0.05; Table 2). In the group of age > 45, none of the *S100P* CpG sites was significantly correlated with BC, and four of the *S100P* CpG sites even had ORs > 1.00 per −10% of methylation, which indicated a potential risk for BC (Table 2).

**TABLE 2:** Methylation differences of *S100P* between BC cases and controls in Study I stratify by median of age.

**Age**	**CpG sites**	**Controls median (IQR)**	**BC cases median (IQR)**	OR (95% CI)* per −10% methylation	*p*-value*
Age ≤ 45 (case = 144 Control = 192)	S100P_CpG_2.3	0.63 (0.59–0.66)	0.64 (0.61–0.68)	0.46 (0.24–0.86)	**0.016**
	S100P_CpG_4	0.69 (0.66–0.74)	0.71 (0.68–0.76)	0.68 (0.42–1.12)	0.130
	S100P_CpG_7	0.40 (0.35–0.46)	0.41 (0.34–0.48)	0.81 (0.61–1.08)	0.157
	S100P_CpG_8	0.46 (0.41–0.51)	0.48 (0.42–0.54)	0.85 (0.60–1.19)	0.343
	S100P_CpG_9	0.49 (0.42–0.56)	0.49 (0.44–0.55)	0.61 (0.41–0.91)	**0.014**
	S100P_CpG_10.11.12	0.61 (0.57–0.64)	0.64 (0.58–0.67)	0.55 (0.35–0.88)	**0.012**
Age > 45 (case = 143 Control = 140)	S100P_CpG_2.3	0.65 (0.62–0.69)	0.67 (0.62–0.70)	1.39 (0.58–3.34)	0.458
	S100P_CpG_4	0.72 (0.68–0.76)	0.71 (0.68–0.76)	0.76 (0.40–1.46)	0.407
	S100P_CpG_7	0.43 (0.36–0.51)	0.42 (0.35–0.46)	1.33 (0.89–1.99)	0.163
	S100P_CpG_8	0.50 (0.44–0.54)	0.51 (0.43–0.57)	1.49 (0.93–2.39)	0.102
	S100P_CpG_9	0.55 (0.46–0.59)	0.58 (0.48–0.64)	0.77 (0.44–1.35)	0.358
	S100P_CpG_10.11.12	0.63 (0.60–0.67)	0.64 (0.61–0.68)	0.89 (0.48–1.66)	0.717

*Logistic regression, adjusted for age and batches of measurement. BC, breast cancer; S100P, S100 calcium-binding protein P gene; OR, odds ratio; IQR, interquartile range. The bold values indicate p < 0.05.

Interestingly, all the four CpG sites of *HYAL2* had ORs > 1.20 per −10% of methylation in the group of older than 45 years. Three were significantly correlated with BC (per −10% of methylation, OR = 2.25 for HYAL2_CpG_1, OR = 1.98 for HYAL2_CpG_2, and OR = 2.25 for HYAL2_CpG_3; all *p* < 0.05; Table 3). In contrast, all the four *HYAL2* CpG sites had ORs < 1.00 per −10% of methylation in the group of younger than 45 years, and none was significant (Table 3).

**TABLE 3:** Methylation differences of *HYAL2* between BC cases and controls in Study I stratify by median of age.

**Age**	**CpG sites**	**Controls median (IQR)**	**BC cases median (IQR)**	OR (95% CI)* per −10% methylation	*p*-value*
Age ≤ 45 (case = 144 Control = 192)	HYAL2_CpG_1	0.32 (0.28–0.36)	0.32 (0.28–0.37)	0.93 (0.61–1.43)	0.751
	HYAL2_CpG_2	0.23 (0.19–0.27)	0.24 (0.19–0.31)	0.85 (0.59–1.22)	0.370
	HYAL2_CpG_3	0.37 (0.34–0.41)	0.37 (0.33–0.42)	0.97 (0.58–1.61)	0.895
	HYAL2_CpG_4	0.53 (0.48–0.57)	0.54 (0.48–0.57)	0.87 (0.59–1.28)	0.477
Age > 45 (case = 143 Control = 140)	HYAL2_CpG_1	0.34 (0.30–0.37)	0.32 (0.28–0.37)	2.25 (1.04–4.85)	**0.039**
	HYAL2_CpG_2	0.24 (0.20–0.30)	0.22 (0.16–0.27)	1.98 (1.12–3.51)	**0.018**
	HYAL2_CpG_3	0.39 (0.35–0.42)	0.37 (0.31–0.41)	2.25 (1.02–4.94)	**0.043**
	HYAL2_CpG_4	0.54 (0.50–0.59)	0.54 (0.47–0.60)	1.23 (0.67–2.49)	0.452

*Logistic regression, adjusted for age and batches of measurement. BC, breast cancer; HYAL2, hyaluronoglucosaminidase 2 gene; OR, odds ratio; IQR, interquartile range. The bold values indicate p < 0.05.

### Breast Cancer-Associated Hypomethylation of S100P and HYAL2 in Study II With Elder People

**FIGURE 2:**
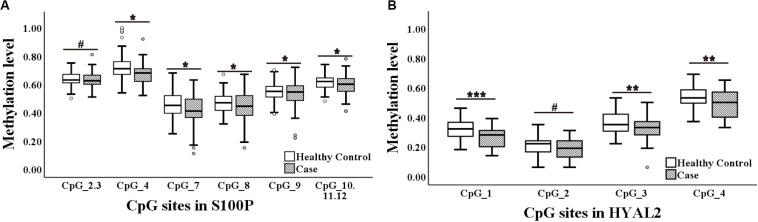
Methylation levels of CpG sites in S100 calcium-binding protein P gene (*S100P*) and hyaluronoglucosaminidase 2 gene (*HYAL2*) in sporadic breast cancer (BC) cases and controls in Study II measured by MassARRAY. **(A)** Box plot shows the distribution of methylation levels of all nine measured CpG sites in *S100P*. **(B)** Box plot shows the distribution of methylation levels of all four measured CpG sites in *HYAL2*. The *p*-values were calculated by logistic regression adjusted for age and batches of measurement; ^#^*p* > 0.05, *p ≤ 0.05, **p ≤ 0.01, ***p ≤ 0.001.

Since both *S100P* and *HYAL2* showed age-related correlation with BC, we further collected blood samples from women with older age. Study II contained 64 BC samples from West China Hospital with the median age of 52.5 years old and 95 cancer-free female controls from the Health Center in Jiangsu Province Hospital of Chinese Medicine. As presented in Figure 2 and Table 4, hypomethylation of seven out of nine CpG sites of *S100P* and three out of four CpG sites of *HYAL2* were significantly correlated with BC by logistic regression adjusted for age and batches of measurements (seven *S100P* CpG sites: ORs from 1.54 to 1.90, *p* < 0.03 for all; three *HYAL2* CpG sites: ORs from 1.97 to 2.49, *p* < 0.01 for all; Table 4). These results proved our hypotheses that hypomethylation of *S100P* and *HYAL2* is correlated with BC in women with older age and were consistent with the previously reported BC-associated *S100P* and *HYAL2* hypomethylation in Caucasians who are mostly above 50 years old.

**TABLE 4:** Methylation differences of *S100P* and *HYAL2* between BC cases and controls in Study II.

**CpG sites**	**Controls median (IQR)**	**BC cases median (IQR)**	OR (95% CI)* per −10% methylation	*p*-value*
S100P_CpG_2.3	0.63 (0.61–0.67)	0.62 (0.60–0.66)	1.38 (0.71–2.69)	0.348
S100P_CpG_4	0.71 (0.67–0.76)	0.68 (0.60–0.71)	1.90 (1.23–2.95)	**0.004**
S100P_CpG_7	0.45 (0.39–0.52)	0.41 (0.35–0.49)	1.54 (1.11–2.15)	**0.011**
S100P_CpG_8	0.46 (0.41–0.51)	0.44 (0.36–0.50)	1.64 (1.09–2.47)	**0.017**
S100P_CpG_9	0.55 (0.51–0.58)	0.54 (0.48–0.59)	1.66 (1.06–2.60)	**0.028**
S100P_CpG_10.11.12	0.62 (0.58–0.65)	0.59 (0.54–0.64)	1.87 (1.07–3.29)	**0.029**
HYAL2_CpG_1	0.32 (0.27–0.36)	0.29 (0.22–0.32)	2.49 (1.42–4.34)	**0.001**
HYAL2_CpG_2	0.22 (0.18–0.26)	0.21 (0.14–0.24)	1.61 (0.93–2.77)	0.086
HYAL2_CpG_3	0.36 (0.31–0.40)	0.34 (0.28–0.38)	1.97 (1.18–3.30)	**0.009**
HYAL2_CpG_4	0.54 (0.51–0.59)	0.52 (0.42–0.57)	2.04 (1.30–3.22)	**0.002**

*Logistic regression, adjusted for age and batches of measurement. BC, breast cancer; S100P, S100 calcium-binding protein P gene; HYAL2, hyaluronoglucosaminidase 2 gene; OR, odds ratio; IQR, interquartile range. The bold values indicate p < 0.05.

Due to the limited sample size of each study, the subjects from the two studies were analyzed together for better estimation. With a total of 349 sporadic BC cases and 427 controls, altered methylation of *S100P* and *HYAL2* showed significant association with BC (Supplementary Table 4), especially for women above the median of age (Supplementary Table 5). In the combined analyses, the association between hypomethylation of *HYAL2* and BC was rather robust, whereas the association between *S100P* and BC was possibly varied among CpGs (Supplementary Tables 4, 5).

### Association Between Altered Methylation in S100P and HYAL2 and the Clinical Characteristics of Breast Cancer

Taking BC cases of the two studies together, the relationship between blood-based *S100P* and *HYAL2* methylation and the clinical characteristics of BC was investigated. Patients with larger BC tumors had lower methylation levels in S100P_CpG_4, S100P_CpG_8, and S100P_CpG_9 (*p* < 0.05; Supplementary Table 6), and patients with positive human epidermal growth factor receptor 2 (HER2) receptors showed lower methylation levels in S100P_CpG_4 and S100P_CpG_10,11,12 (*p* < 0.05; Supplementary Table 6). In *HYAL2*, hypomethylation of HYAL2_CpG_1 was associated with estrogen receptor (ER)-positive status and non-triple-negative BC (*p* < 0.01; Supplementary Table 7). Other CpG sites in *S100P* and *HYAL2* showed no or rather borderline associations with clinical characteristics of BC.

## Discussion

Although several studies have reported BC-related methylation in peripheral blood in the Caucasian population, few studies were carried out in Asia. In our previous study, we reported the blood-based hypomethylation of *S100P* and *HYAL2* as a risk factor for BC in the Caucasian population (Yang et al., 2015, 2017). Here, we investigate the associations between BC and DNA methylation of *S100P* and *HYAL2* in the Chinese population in two independent case-control studies with a total of 778 subjects. In our study, we were surprised to observe an age-dependent association between BC and methylation levels of *S100P* and *HYAL2* in peripheral blood. Partly in agreement with the observation in Caucasians (Yang et al., 2015, 2017), hypomethylation of *S100P* and *HYAL2* was a risk factor for BC, but only for elder Chinese women, specifically for women older than 50 years. For women younger than 45 years, hypomethylation of *S100P* showed a protective effect for BC, whereas *HYAL2* methylation was not correlated.

To understand the differential DNA methylation patterns between ethnicities, we took a closer look at the DNA methylation loci in *S100P* and *HYAL2*. The methylation levels of *S100P* and *HYAL2* CpG sites in healthy Chinese individuals are all lower than that of healthy Europeans (Yang et al., 2015, 2017), but the methylation levels are mostly similar in BC cases of the two ethnic groups. For some loci, the levels of methylation differed remarkably; the methylation level of S100P_CpG_4 in healthy Chinese individuals is 22% lower than that of Caucasians, whereas the methylation level of HYAL2_CpG_2 in Chinese BC cases is around 25% higher than that in European BC patients. Therefore, we only observed a weak correlation between the methylation of *S100P* and *HYAL2* in the Chinese population, and mostly in the women above 50. Moreover, S100P_CpG_4, which had no correlation with BC in Caucasians, showed significance in the Chinese population, whereas HYAL2_CpG_2 lost its association with BC in the Chinese population. Variant white blood cell compositions in different ethnic groups can be one of the reasons for the altered methylation between Chinese and Europeans. Unfortunately, there is so far no report about the white blood cell composition difference between Chinese and Europeans. But African Americans and European Americans did have differences in white blood cell counts (Nalls et al., 2008). Nevertheless, the change of white blood cell composition cannot explain why only two CpG sites, S100P_CpG_4 and HYAL2_CpG_2, showed dramatic methylation differences between Chinese and Europeans. Zhang et al. (2011) also observed significant differences in global genomic DNA methylation by ethnicity in peripheral blood. Elliott et al. (2014) further reported differences in smoking-associated DNA methylation patterns in South Asians and Europeans and concluded that there is a true ethnic difference in methylation signatures. On the other hand, lifestyle may contribute to DNA methylation as well. One study in native Japanese and Japanese-American men sharing similar genetic backgrounds disclosed significant differences in body compositions due to altered environments or lifestyles and consequently have a variant influence to carcinogenesis (Marks et al., 2004). Several studies also suggested the influence of population, temperature, humidity, diet, and nutrition on DNA methylation (Bind et al., 2014; Panni et al., 2016; Sapienza and Issa, 2016; Martin and Fry, 2018). Taken together, genetic background and different lifestyles can be confounders for *S100P*- and *HYAL2*-associated BC risk in different ethnicities.

The major mechanism for the epigenetic related hereditary background is the differential genetic variations and frequencies in different populations. Genetic features are one of the major factors for BC. So far, there are no studies about the variations of *S100P* and *HYAL2* in different ethnic groups. Nevertheless, the methylation/expression of *S100P* and *HYAL2* is regulated by multiple genes; we could assume that the upstream genetic variations may modulate the regulation of *S100P* and *HYAL2*. In humans, the S100 protein family is composed of 21 members, which modulates cellular responses by functioning both as intracellular Ca^2+^ sensors and as extracellular factors and further actively contribute to tumorigenic processes, such as cell proliferation, metastasis, angiogenesis, and immune evasion (Arumugam et al., 2004; Jiang et al., 2012; Bresnick et al., 2015). S100 family proteins could also modulate the tumor microenvironment and guide the trafficking of leukocytes via the interactions with inflammation chemokines (Nasser et al., 2015). As one of the S100 proteins family, S100P is involved in and interacts with multiple pathways, such as mitogen-activated protein kinase (MAPK) and nuclear factor (NF)-κB pathway, Ras GTPase pathways, receptor for advanced glycation end products (RAGE) pathways, etc. (Arumugam et al., 2004; Jiang et al., 2012; Bresnick et al., 2015). In different ethnic groups, variant mutation frequencies have been observed in the MAPK pathway, like *BRAF*, *NRAS*, *C-KIT*, and *PDGFRA*, as well *TERT* (Si et al., 2012; Bai et al., 2017). *HYAL2*, one of the enzymes that degrade hyaluronan (HA), is a tumor suppressor gene involved in cancer progression, angiogenesis, metastasis, chemokinesis, cell adhesion, and cell mobility (Hesson et al., 2007; Saito et al., 2011). The expression of *HYAL2* could be regulated by cytokines and growth factors, such as tumor necrosis factor (TNF)-α, interleukin (IL)-1β (Monzon et al., 2008), and transforming growth factor (TGF)-beta (Hsu et al., 2009), which also show ethnic differences in gene polymorphisms (Baena et al., 2002; Niu et al., 2012). Therefore, regulated by multiple genes, the epigenetic modification and expression of *S100P* and *HYAL2* might be affected by the population-based genetic variations and frequencies.

In Caucasians whose BC was diagnosed in the average age of 56, blood-based hypomethylation of *S100P* and *HYAL2* was a risk factor (Yang et al., 2015, 2017). In Chinese, the average age for BC diagnosis is only 45 years old (Li et al., 2016). The mean age of menopause in Asia is 48.8 years (Dunneram et al., 2019), and the perimenopausal period is normally 3–4 years before menopause which means 45–49 years. Thus, below 45 years could be considered as premenopausal, and above 45 years are mostly perimenopausal and postmenopausal. Here, we found an age or menopause status-related association between BC and methylation levels of *S100P* and *HYAL2* in peripheral blood. Studies have shown that Asian babies are exposed to a higher level of estrogens before birth than Caucasians by measuring the umbilical cord blood plasma (Shibata et al., 2002), and the young Japanese women have a higher level of 17 beta-estradiol than Caucasian women (Hill et al., 1976). Therefore, the earlier incidence age of BC in Chinese might be due to the higher exposure level of sex hormones at a younger age. The transcriptional regulation of *S100P* expression is highly dependent on the type of cancer and tissue. The expression of *S100P* in the colon is mediated by prostaglandin E2 (PGE2)–prostaglandin E receptor 4 (PTGER4) signaling (Chandramouli et al., 2010), in prostate cancer by IL-6 (Hammacher et al., 2005), and in breast and cervical by glucocorticoids (Tothova et al., 2011). More specifically, the expression of *S100P* could be influenced by sex steroids in the system. Zhang et al. (2012) reported that progesterone could dramatically upregulate the expression of *S100P* in both primary endometrial epithelial and stromal cells. In BC cell lines, seven progenies could regulate the expression of *S100P* (Bray et al., 2005). Probably due to the interaction between *S100P* expression and sex steroids, lower expression of *S100P* was associated with poor survival of ovarian cancer (Umezaki et al., 2015). Estrogen is an upstream signal that diversely regulates the expression of 3p21.3 genes, including *HYAL1* and *HYAL2* (Edjekouane et al., 2016). Meanwhile, glucocorticoids could increase the expression of HA synthases and reduced the expression of hyaluronidases (Papakonstantinou et al., 2012). The methylation of *S100P* and *HYAL2* has been proofed to be inversely correlated with the expression in leukocytes (Yang et al., 2015, 2017). Thus, we may assume that the methylation/expression of *S100P* and *HYAL2* in the circulating leukocytes might be influenced by the sex hormone levels in the blood.

In our study, we also observed slightly lower *S100P* methylation in patients with larger BC tumors and patients with positive HER2 receptors; lower *HYAL2* methylation in patients with ER-positive and non-triple-negative tumor. But these effects were weak and need further validation in larger studies. Nevertheless, the similar methylation levels between BC patients with different clinical characteristics further suggested that the aberrant methylation of *S100P* and *HYAL2* could be applied for the detection of BC in general regardless of subtype and clinical status.

In conclusion, this study provided further evidence for the association between altered methylation of *S100P* and *HYAL2* and BC, which is mostly for postmenopausal women. We also suggested the influence of genetic background, lifestyle, and hormone status as confounders for DNA methylation. The BC cases of Study I were from Northeast China (Beijing), whereas the BC cases of Study II are from Southwest China (Chengdu). The lifestyle and diet vary a lot from Northeast to Southwest of China. The combined analyses might be influenced by these covariants, which may influence the methylation levels of *S100P* but not much on *HYAL2*. Surely, the inconsistency may also be due to limited sample size, and thus, validation in multicenter studies with enlarged sample sizes is necessary. Therefore, we highlighted that the epigenetic biomarkers in one ethnic group warrant population-based validation when its application in another ethnic group is considered.

## Data Availability Statement

The raw data supporting the conclusions of this article will be made available by the authors, without undue reservation, to any qualified researcher.

## Ethics Statement

The studies involving human participants were reviewed and approved by the Ethics Committee of Nanjing Medical University, the Ethics Committee of Chinese Academy of Medical Sciences, and the Ethics Committee of West China Hospital. The patients/participants provided their written informed consent to participate in this study.

## Author Contributions

RY and QY designed the experiment and wrote the manuscript. QY performed all the experiments and analyzed the results. XY, LL, TX, WZ, WG, and FM provided the materials and supervised the patient enrollment and acquisition of biological samples and clinical data. All authors contributed to the article and approved the submitted version.

## Conflict of Interest

The authors declare that the research was conducted in the absence of any commercial or financial relationships that could be construed as a potential conflict of interest.

## Abbreviations

BC: Breast cancer
ER: Estrogen receptor
HA: Hyaluronan
HER2: Human epidermal growth factor receptor 2
*HYAL2*: Hyaluronoglucosaminidase 2 gene
OR: Odds ratio
PCR: Polymerase chain reaction
PR: Progesterone receptor
MALDI-TOF: Matrix-assisted laser desorption/ionization-time-of-flight
*S100P*: S100 calcium-binding protein P gene.
