# Supplementary material for: The Association Between Breast Cancer and Blood-Based Methylation of S100P and HYAL2 in the Chinese Population
Source: Front Genet. 2020 Aug 28;11:977. doi: 10.3389/fgene.2020.00977 (PMC7485126; doi:10.3389/fgene.2020.00977)
Supplement: Supplementary file 1 [file Data_Sheet_1.docx]

**Supplementary Figures**

**Supplementary Figure 1**

**A**

GGCAGTGCTGCCCACGGCCATAGAGAGCCCTGAGTGAGGAGAGATCTGGGGAGTTCTGGGCCGTCCACCGGCAAAGGACAATGGATCTCCAAGGGGTGCGGCCAGCGGATGGGTGATGGCATGGATGGAGGGTGGGTGCATCAATGCTGGAGCATCAACTCTGTGTGCCCCTGCGGGGAGAATCGCCCCTGCCGAAGCCCCGGCTGGTCCAAAACCACGCCAGCCGCTTTAGGGGTGTCTGTGGACCCAGGACCACACAGCCTCAAGAGCCAAGAGTCCCGAGTGTG

**B**

TTTTTTTTTTTTTGAGACAGAGTCTTGCTCTCTCATCCAGGCTGGAATGCAGTGGCCCAGTCTCGACTCACTGCAACCTCTGTCTTCTGGGTTCAAGTGATACTCCTGCCTCAGCCTCCCCAGTAGCTGGGATTACAGGCACACACCACCATGGCCAGATAATTTTTTTGTATTTTCAGTACAGACGGGGTTTTGCTATGTTGGCCTGGCAGGTCTCGAACTCTTGGCCTCAAGTGATCTGCCTGCCTTGGCCTCCCAAAATGCTGAGATTACAGGCATGAGTCACCAAGCCCAGCCTTCTTTCTTTTTTTTGAGACAGAGCCTCACCCTGTCACCCAGGTTGGAGTGCAGTGGCACGATCTTGGCTCACTGCAACCTTTGCCTCCCGGTTGAAGTGATTCAGTCTCCCAAGTAGCTGGGACTACAGTCACACACCACCATGCCCGGCTAATTTTTGTATGTTTAGTAGAGATAGGGTTTCACCATGTTGGCCAGGCTGACCTCGAATTCCTGATTGCAAATGATCCACCTGCCTTGGCCTCCCAAAGCATTGGCATTAGAGGTGTGA

**Supplementary Figure 1 A**: Sequences of *S100P* amplicon for MassARRAY methylation analysis (Chr4: 6746537 - 6746823, build hg38, defined by the UCSC Genome Browser). **B**: Sequences of *HYAL2* amplicon for MassARRAY methylation analysis (Chr3: 50335322 - 50335889, build hg38, defined by the UCSC Genome Browser). The MassARRAY assay determined the methylation levels of 13 CpGs and yielded 10 distinguishable peaks.

**Supplementary Tables**

**Supplementary Table 1**: Inter-quartile analysis for *S100P* methylation level between BC cases and controls in Study I

| CpG sites | Quartiles (methylation range) | Controls N (%) | BC cases N (%) | Total | OR (95%CI) | *p*-value ***** | *p*_trend_ |
| --- | --- | --- | --- | --- | --- | --- | --- |
| S100P_CpG_2.3 | Q4 (> 0.68) | 63 (19.1) | 77 (27.2) | 140 | 1 | - | **0.003** |
|  | Q3 (≤ 0.68) | 83 (25.2) | 79 (27.9) | 162 | 0.77 (0.49 - 1.22) | 0.268 |  |
|  | Q2 (≤ 0.65) | 77 (23.3) | 58 (20.5) | 135 | 0.61 (0.38 - 0.99) | **0.044** |  |
|  | Q1 (≤ 0.61) | 107 (32.4) | 69 (24.4) | 176 | 0.52 (0.33 - 0.83) | **0.005** |  |
| S100P_CpG_4 | Q4 (> 0.75) | 79 (24.0) | 74 (26.1) | 153 | 1 | - | 0.124 |
|  | Q3 (≤ 0.75) | 63 (19.1) | 65 (23.0) | 128 | 1.10 (0.69 - 1.77) | 0.681 |  |
|  | Q2 (≤ 0.71) | 93 (28.3) | 80 (28.3) | 173 | 0.92 (0.60 - 1.42) | 0.710 |  |
|  | Q1 (≤ 0.67) | 94 (28.6) | 64 (22.6) | 158 | 0.73 (0.47 - 1.15) | 0.172 |  |
| S100P_CpG_7 | Q4 (> 0.48) | 77 (23.3) | 62 (21.9) | 139 | 1 | - | 0.188 |
|  | Q3 (≤ 0.48) | 79 (23.9) | 65 (23.0) | 144 | 1.03 (0.64 - 1.64) | 0.918 |  |
|  | Q2 (≤ 0.42) | 88 (26.7) | 72 (25.4) | 160 | 1.03 (0.65 - 1.62) | 0.915 |  |
|  | Q1 (≤ 0.35) | 86 (26.1) | 84 (29.7) | 170 | 1.22 (0.78 - 1.92) | 0.385 |  |
| S100P_CpG_8 | Q4 (> 0.54) | 58 (17.6) | 83 (29.3) | 141 | 1 | - | 0.061 |
|  | Q3 (≤ 0.54) | 97 (29.4) | 66 (23.3) | 163 | 0.48 (0.30 - 0.75) | **0.002** |  |
|  | Q2 (≤ 0.49) | 92 (27.9) | 61 (21.6) | 153 | 0.46 (0.29 - 0.74) | **0.001** |  |
|  | Q1 (≤ 0.42) | 83 (25.2) | 73 (25.8) | 156 | 0.61 (0.39 - 0.98) | **0.041** |  |
| S100P_CpG_9 | Q4 (> 0.59) | 62 (18.8) | 84 (29.7) | 146 | 1 | - | **0.042** |
|  | Q3 (≤ 0.59) | 92 (27.9) | 57 (20.1) | 149 | 0.46 (0.29 - 0.73) | **0.001** |  |
|  | Q2 (≤ 0.52) | 78 (23.6) | 71 (25.1) | 149 | 0.67 (0.42 - 1.06) | 0.089 |  |
|  | Q1 (≤ 0.44) | 98 (29.7) | 71 (25.1) | 169 | 0.53 (0.33 - 0.84) | **0.007** |  |
| S100P_CpG_10.11.12 | Q4 (> 0.66) | 62 (18.8) | 91 (32.2) | 153 | 1 | - | **2.00E-4** |
|  | Q3 (≤ 0.66) | 71 (21.5) | 59 (20.8) | 130 | 0.57 (0.35 - 0.91) | **0.018** |  |
|  | Q2 (≤ 0.63) | 93 (28.2) | 66 (23.3) | 159 | 0.48 (0.31 - 0.76) | **0.020** |  |
|  | Q1 (≤ 0.59) | 105 (31.5) | 67 (23.7) | 171 | 0.43 (0.28 - 0.68) | **3.00E-4** |  |
| * Logistic regression, adjusted for age and batches of the measurement. | | | | | | | |

**Supplementary Table 2**: Inter-quartile analysis for *HYAL2* methylation between BC cases and controls in Study I

| CpG sites | Quartiles (methylation range) | Controls N (%) | BC cases N (%) | Total | OR (95%CI) | *p*-value***** | *p*_trend_ |
| --- | --- | --- | --- | --- | --- | --- | --- |
| HYAL2_CpG_1 | Q4 (> 0.37) | 61 (18.9) | 67 (23.3) | 128 | 1 | - | 0.611 |
|  | Q3 (≤ 0.37) | 96 (29.8) | 57 (19.9) | 153 | 0.54 (0.34 - 0.87) | **0.011** |  |
|  | Q2 (≤ 0.33) | 86 (26.7) | 83 (28.9) | 169 | 0.88 (0.55 - 1.39) | 0.578 |  |
|  | Q1 (≤ 0.28) | 79 (24.5) | 80 (27.9) | 159 | 0.92 (0.58 - 1.47) | 0.724 |  |
| HYAL2_CpG_2 | Q4 (> 0.28) | 76 (23.6) | 72 (25.1) | 148 | 1 |  | 0.314 |
|  | Q3 (≤ 0.28) | 87 (27.0) | 63 (22.0) | 150 | 0.77 (0.48 - 1.21) | 0.251 |  |
|  | Q2 (≤ 0.23) | 78 (24.2) | 59 (20.6) | 137 | 0.80 (0.50 - 1.28) | 0.349 |  |
|  | Q1(≤ 0.19) | 81 (25.2) | 93 (32.4) | 174 | 1.21 (0.78 - 1.88) | 0.391 |  |
| HYAL2_CpG_3 | Q4 (> 0.41) | 76 (23.8) | 68 (23.7) | 144 | 1 |  | 0.119 |
|  | Q3 (≤ 0.41) | 87 (27.2) | 63 (22.0) | 150 | 0.81 (0.51 - 1.28) | 0.368 |  |
|  | Q2 (≤ 0.37) | 84 (26.3) | 65 (22.6) | 149 | 0.87 (0.55 - 1.37) | 0.536 |  |
|  | Q1 (≤ 0.33) | 73 (22.8) | 91 (31.7) | 164 | 1.39 (0.89 - 2.19) | 0.150 |  |
| HYAL2_CpG_4 | Q4 (> 0.58) | 74 (23.0) | 67 (23.2) | 141 | 1 |  | 0.842 |
|  | Q3 (≤ 0.58) | 80 (24.8) | 81 (28.0) | 161 | 1.12 (0.71 - 1.76) | 0.622 |  |
|  | Q2 (≤ 0.54) | 95 (29.5) | 57 (20.4) | 152 | 0.67 (0.42 - 1.06) | 0.087 |  |
|  | Q1 (≤ 0.48) | 73 (22.7) | 82 (28.4) | 155 | 1.25 (0.79 - 1.97) | 0.347 |  |
| * Logistic regression, adjusted for age and batches of the measurement. | | | | | | | |

**Supplementary Table 3**：Correlation between age and DNA methylation levels in controls and BC cases respectively in Study I

| CpG sites | Age and methylation levels in controls | | Age and methylation levels in BC cases | |
| --- | --- | --- | --- | --- |
|  | Spearman rho | *p*-value | Spearman rho | *p*-value |
| S100P_CpG_2.3 | 0.241 | **9.00E-6** | 0.257 | **1.20E-5** |
| S100P_CpG_4 | 0.161 | **0.003** | 0.067 | 0.263 |
| S100P_CpG_7 | 0.150 | **0.006** | 0.042 | 0.478 |
| S100P_CpG_8 | 0.229 | **2.70E-5** | 0.192 | **0.001** |
| S100P_CpG_9 | 0.244 | **7.00E-6** | 0.375 | **7.30E-11** |
| S100P_CpG_10.11.12 | 0.179 | **0.001** | 0.133 | **0.025** |
| HYAL2_CpG_1 | 0.027 | 0.629 | -0.024 | 0.688 |
| HYAL2_CpG_2 | 0.079 | 0.160 | -0.146 | **0.013** |
| HYAL2_CpG_3 | -0.045 | 0.418 | -0.132 | **0.025** |
| HYAL2_CpG_4 | 0.117 | **0.037** | 0.054 | 0.358 |

| CpG sites | Controls  median (IQR) | Cases  median (IQR) | OR (95% CI) **^*^** per -10% methylation | *p*-value **^*^** |
| --- | --- | --- | --- | --- |
| S100P_CpG_2.3 | 0.64 (0.61 – 0.67) | 0.65 (0.61 – 0.69) | 0.58 (0.43 – 0.79) | **0.001** |
| S100P_CpG_4 | 0.71 (0.67 – 0.75) | 0.71 (0.67 – 0.75) | 1.18 (0.95 – 1.45) | 0.135 |
| S100P_CpG_7 | 0.42 (0.36 – 0.49) | 0.41 (0.35 – 0.47) | 1.39 (1.18 – 1.64) | **9.60E-5** |
| S100P_CpG_8 | 0.48 (0.42 – 0.52) | 0.48 (0.42 – 0.55) | 0.85 (0.71 – 1.01) | 0.067 |
| S100P_CpG_9 | 0.53 (0.45 – 0.58) | 0.53 (0.45 – 0.60) | 0.80 (0.68 – 0.93) | **0.005** |
| S100P_CpG_10.11.12 | 0.62 (0.58 – 0.65) | 0.63 (0.58 – 0.67) | 0.79 (0.62 – 1.01) | 0.061 |
| HYAL2_CpG_1 | 0.33 (0.28 – 0.36) | 0.31 (0.27 – 0.36) | 1.23 (0.99 – 1.55) | 0.068 |
| HYAL2_CpG_2 | 0.23 (0.19 – 0.27) | 0.23 (0.17 – 0.18) | 1.11 (0.90 – 1.37) | 0.344 |
| HYAL2_CpG_3 | 0.37 (0.33 – 0.41) | 0.36 (0.31 – 0.41) | 1.58 (1.25 – 2.01) | **1.64E-4** |
| HYAL2_CpG_4 | 0.53 (0.49 – 0.58) | 0.53 (0.47 – 0.58) | 1.40 (1.14 – 1.73) | **0.001** |
| **^*^** Logistic regression, adjusted for age and batches of measurement. | | | | |

**Supplementary Table 4**: Methylation differences of *S100P* and *HYAL2* between BC cases and controls in Study I & Ⅱ

**Supplementary Table 5**: Methylation differences of *S100P* and *HYAL2* between BC cases and controls in Study I & II stratify by 45 years old^§^

| Age | CpG sites | Controls  median (IQR) | BC Cases  median (IQR) | OR (95% CI) *  per -10% methylation | *p***-**value***** |
| --- | --- | --- | --- | --- | --- |
| Age ≤ 45  (case = 161  Control = 235) | S100P_CpG_2.3 | 0.64 (0.60 – 0.67) | 0.64 (0.61 – 0.68) | 0.57 (0.35 – 0.92) | **0.021** |
|  | S100P_CpG_4 | 0.71 (0.66 – 0.75) | 0.71 (0.68 – 0.75) | 1.00 (0.72 – 1.39) | 0.986 |
|  | S100P_CpG_7 | 0.42 (0.35 – 0.48) | 0.41 (0.34 – 0.48) | 1.44 (1.11 – 1.87) | **0.006** |
|  | S100P_CpG_8 | 0.47 (0.41 – 0.52) | 0.47 (0.42 – 0.52) | 0.88 (0.66 – 1.16) | 0.365 |
|  | S100P_CpG_9 | 0.52 (0.44 – 0.57) | 0.49 (0.44 – 0.55) | 0.99 (0.77 – 1.29) | 0.964 |
|  | S100P_CpG_10.11.12 | 0.62 (0.58 – 0.65) | 0.63 (0.58 – 0.67) | 0.82 (0.56 – 1.20) | 0.314 |
|  | HYAL2_CpG_1 | 0.32 (0.27 – 0.36) | 0.32 (0.27 – 0.37) | 1.11 (0.76 – 1.60) | 0.597 |
|  | HYAL2_CpG_2 | 0.23 (0.19 – 0.27) | 0.24 (0.19 – 0.31) | 0.72 (0.52 – 1.01) | 0.058 |
|  | HYAL2_CpG_3 | 0.37 (0.33 – 0.41) | 0.37 (0.32 – 0.42) | 1.18 (0.79 – 1.75) | 0.416 |
|  | HYAL2_CpG_4 | 0.53 (0.49 – 0.58) | 0.53 (0.48 – 0.57) | 1.42 (1.01 – 1.99) | **0.041** |
| Age > 45  (case = 188  Control = 192) | S100P_CpG_2.3 | 0.64 (0.61 – 0.68) | 0.66 (0.61 – 0.69) | 0.58 (0.38 – 0.88) | **0.011** |
|  | S100P_CpG_4 | 0.71 (0.67 – 0.76) | 0.71 (0.66 – 0.75) | 1.38 (1.02 – 1.860 | **0.037** |
|  | S100P_CpG_7 | 0.44 (0.37 – 0.50) | 0.41 (0.35 – 0.47) | 1.39 (1.11 – 1.74) | **0.004** |
|  | S100P_CpG_8 | 0.49 (0.43 - 0.53) | 0.49 (0.41 – 0.56) | 0.85 (0.66 – 1.08) | 0.187 |
|  | S100P_CpG_9 | 0.54 (0.44 – 0.59) | 0.57 (0.48 – 0.63) | 0.66 (0.53 – 0.82) | **1.64E-4** |
|  | S100P_CpG_10.11.12 | 0.62 (0.58 – 0.65) | 0.63 (0.59 – 0.67) | 0.85 (0.60 – 1.21) | 0.375 |
|  | HYAL2_CpG_1 | 0.34 (0.29 - 0.37) | 0.31 (0.27 – 0.36) | 1.49 (1.09 – 2.04) | **0.012** |
|  | HYAL2_CpG_2 | 0.24 (0.20 – 0.28) | 0.22 (0.16 – 0.26) | 1.75 (1.28 – 2.38) | **4.35E-4** |
|  | HYAL2_CpG_3 | 0.38 (0.34 – 0.41) | 0.36 (0.31 – 0.40) | 2.02 (1.46 – 2.81) | **2.50E-5** |
|  | HYAL2_CpG_4 | 0.54 (0.50 – 0.59) | 0.54 (0.47 – 0.59) | 1.51 (1.14 – 2.01) | **0.004** |
| * Logistic regression, adjusted for age and batches of measurement. | | | | | |
| ^§^ The age of two BC cases in study II is missing, and thus a total of 249 cases was included in this study. | | | | | |

**Supplementary Table 6:** The association between *S100P* methylation level and the clinical characteristics of BC patients

| Clinical characteristics | Group (N) | Median of age | Median of methylation levels in the CpG sites of *S100P* | | | | | |
| --- | --- | --- | --- | --- | --- | --- | --- | --- |
|  |  |  | S100P_CpG_2.3 | S100P_CpG_4 | S100P_CpG_7 | S100P_CpG_8 | S100P_CpG_9 | S100P_CpG_10.11.12 |
| Tumor stage (313) | Stage 0&I (142) | 46.0 | 0.65 | 0.71 | 0.42 | 0.50 | 0.55 | 0.62 |
|  | Stage II (123) | 47.0 | 0.65 | 0.71 | 0.42 | 0.48 | 0.53 | 0.64 |
|  | Stage III&IV (48) | 47.0 | 0.64 | 0.71 | 0.41 | 0.46 | 0.49 | 0.61 |
|  | *p*-value (Kruskal-Wallis test) | 0.467 | 0.306 | 0.713 | 0.742 | 0.250 | 0.058 | 0.731 |
| Tumor size (314) | Tis&T1(189) | 46.0 | 0.65 | 0.71 | 0.42 | 0.50 | 0.55 | 0.64 |
|  | T2(116) | 47.0 | 0.65 | 0.71 | 0.41 | 0.48 | 0.52 | 0.62 |
|  | T3&T4(9) | 43.0 | 0.61 | 0.64 | 0.39 | 0.42 | 0.49 | 0.57 |
|  | *p*-value (Kruskal-Wallis test) | 0.281 | 0.092 | **0.005** | 0.251 | **0.039** | **0.043** | 0.021 |
| Lymph node (LN) involvement (316) | no involved LN (195) | 46.5 | 0.65 | 0.71 | 0.42 | 0.49 | 0.54 | 0.62 |
|  | > 1 involved LN (121) | 46.0 | 0.65 | 0.71 | 0.41 | 0.48 | 0.51 | 0.64 |
|  | *p*-value (Mann-Whitney U) | 0.184 | 0.948 | 0.297 | 0.238 | 0.222 | 0.075 | 0.383 |
| Ki67 (320) ┴ | Low (85) | 47.0 | 0.65 | 0.71 | 0.43 | 0.50 | 0.53 | 0.63 |
|  | High (238) | 46.0 | 0.65 | 0.71 | 0.41 | 0.48 | 0.54 | 0.63 |
|  | *p*-value (Mann-Whitney U) | 0.455 | 0.820 | 0.813 | 0.067 | 0.329 | 0.495 | 0.314 |
| ER status (329) | ER negative (72) | 47.0 | 0.65 | 0.71 | 0.42 | 0.48 | 0.52 | 0.62 |
|  | ER positive (257) | 46.0 | 0.65 | 0.71 | 0.41 | 0.49 | 0.54 | 0.63 |
|  | *p*-value (Mann-Whitney U) | 0.305 | 0.789 | 0.714 | 0.514 | 0.440 | 0.541 | 0.685 |
| PR status (329) | PR negative (97) | 47.0 | 0.65 | 0.71 | 0.42 | 0.48 | 0.54 | 0.62 |
|  | PR positive (232) | 46.0 | 0.65 | 0.71 | 0.41 | 0.48 | 0.54 | 0.63 |
|  | *p*-value (Mann-Whitney U) | 0.200 | 0.501 | 0.610 | 0.883 | 0.793 | 0.994 | 0.843 |
| HER2 status (329) | HER2 negative (215) | 46.0 | 0.66 | 0.71 | 0.41 | 0.49 | 0.52 | 0.63 |
|  | HER2 positive (114) | 48.0 | 0.64 | 0.70 | 0.42 | 0.47 | 0.55 | 0.62 |
|  | *p*-value (Mann-Whitney U) | **0.031** | 0.191 | **0.041** | 0.539 | 0.285 | 0.282 | **0.028** |
| Three receptors  status (328) | Triple-negative (38) | 46.0 | 0.66 | 0.71 | 0.42 | 0.48 | 0.49 | 0.63 |
|  | Non -triple-negative (290) | 45.5 | 0.65 | 0.71 | 0.41 | 0.48 | 0.53 | 0.64 |
|  | *p*-value (Mann-Whitney U) | 0.816 | 0.321 | 0.634 | 0.897 | 0.741 | 0.687 | 0.642 |
| **┴** Individuals with ≥ 20% and < 20% of Ki67 were considered as populations at high and low cell proliferation respectively. Compared their methylation level by non-parametric test. | | | | | | | | |

**Supplementary Table 7:** The association between *HYAL2* methylation level and the clinical characteristics of BC patients

| Clinical characteristics | Group | Median of age | Median of methylation levels in the CpG sites of *HYAL2* | | | |
| --- | --- | --- | --- | --- | --- | --- |
|  |  |  | HYAL2_CpG_1 | HYAL2_CpG_2 | HYAL2_CpG_3 | HYAL2_CpG_4 |
| Tumor stage (313) | Stage 0&I (142) | 46.0 | 0.32 | 0.24 | 0.36 | 0.52 |
|  | Stage II (122) | 47.0 | 0.31 | 0.22 | 0.37 | 0.54 |
|  | Stage III&IV (48) | 47.0 | 0.32 | 0.24 | 0.38 | 0.54 |
|  | *p*-value (Kruskal-Wallis test) | 0.467 | 0.533 | 0.175 | 0.477 | 0.288 |
| Tumor size (314) | Tis&T1 (189) | 46.0 | 0.31 | 0.23 | 0.36 | 0.53 |
|  | T2 (116) | 47.0 | 0.31 | 0.23 | 0.37 | 0.54 |
|  | T3&T4 (9) | 43.0 | 0.32 | 0.26 | 0.37 | 0.52 |
|  | *p*-value (Kruskal-Wallis test) | 0.281 | 0.921 | 0.5536 | 0.808 | 0.970 |
| Lymph node (LN) involvement (316) | no involved LN (195) | 46.5 | 0.31 | 0.23 | 0.36 | 0.52 |
|  | > 1 involved LN (121) | 46.0 | 0.32 | 0.22 | 0.37 | 0.55 |
|  | *p*-value (Mann-Whitney U) | 0.184 | 0.224 | 0.693 | 0.194 | **0.037** |
| Ki67 (323) **┴** | Low (85) | 47.0 | 0.31 | 0.23 | 0.35 | 0.53 |
|  | High (238) | 46.0 | 0.31 | 0.23 | 0.36 | 0.54 |
|  | *p*-value (Mann-Whitney U) | 0455 | 0.671 | 0.744 | 0.673 | 0.386 |
| ER status (329) | ER negative (72) | 47.0 | 0.32 | 0.23 | 0.37 | 0.54 |
|  | ER positive (257) | 46.0 | 0.31 | 0.23 | 0.36 | 0.54 |
|  | *p*-value (Mann-Whitney U) | 0.305 | 0.597 | 0.882 | 0.942 | 0.590 |
| PR status (329) | PR negative (97) | 47.0 | 0.32 | 0.23 | 0.37 | 0.54 |
|  | PR positive (232) | 46.0 | 0.31 | 0.23 | 0.36 | 0.53 |
|  | *p*-value (Mann-Whitney U) | 0.200 | 0.961 | 0.712 | 0.944 | 0.929 |
| HER2 status (329) | HER2 negative (215) | 46.0 | 0.32 | 0.23 | 0.37 | 0.53 |
|  | HER2 positive (114) | 48.0 | 0.30 | 0.22 | 0.36 | 0.54 |
|  | *p*-value (Mann-Whitney U) | **0.031** | **0.002** | 0.302 | 0.165 | 0.707 |
| Three receptors  status (328) | Triple-negative (38) | 46.0 | 0.35 | 0.27 | 0.38 | 0.55 |
|  | Non -triple-negative (290) | 45.5 | 0.31 | 0.23 | 0.36 | 0.53 |
|  | *p*-value (Mann-Whitney U) | 0.816 | **0.010** | **0.049** | 0.133 | 0.142 |
| ┴ Individuals with ≥ 20% and < 20% of Ki67 were considered as populations at high and low cell proliferation respectively. Compared their methylation level by non-parametric test. | | | | | | |
